# Supplementary material for: Morally excused but socially excluded: Denying agency through the defense of mental impairment
Source: PLoS One. 2021 Jun 10;16(6):e0252586. doi: 10.1371/journal.pone.0252586 (PMC8192116; doi:10.1371/journal.pone.0252586)
Supplement: S1 File — (DOCX) [file pone.0252586.s001.docx]

# Supporting Information File for “Morally excused but socially excluded: Denying agency through the defense of mental impairment”

**Contents**

[Study 1 Materials](#_Study_1_Materials)

[Study 2 Materials](#_Study_2_Materials)

[Study 3 Materials (Registered Report)](#_Study_3_Materials)

[Additional Measures in Studies 1 and 2](#_Additional_Measures_in)

[Study 1 Exploratory Analysis: SEM Results](#_Study_1_Exploratory)

# Study 1 Materials

**Manipulation: ControlPre**

Jeff is a 25 year old man, on trial for pushing a stranger off a pedestrian bridge, causing permanent nerve damage and paralysis.   

Jeff enters a guilty plea, and admits full guilt. Jeff is distressed and remorseful about what he has done.

**Manipulation: MIPre**

Jeff is a 25 year old man on trial for pushing a stranger off a pedestrian bridge, causing permanent nerve damage and paralysis. 

Jeff enters an insanity plea. At his trial, expert doctors claim Jeff suffers from ongoing psychosis, caused by schizophrenia.  At the time of the crime, Jeff believed that the stranger was an evil villain, and angels convinced Jeff that he needed to push the man in order to save the world.

The judge rules that Jeff is not guilty by rule of insanity.  Jeff is distressed and remorseful about what he has done.

**Manipulation: ControlPost**

Jeff is a 25 year old man, on trial for pushing a stranger off a pedestrian bridge, causing permanent nerve damage and paralysis.   

Jeff enters a guilty plea, and admits full guilt. Jeff is distressed and remorseful about what he has done.

Jeff is sent to a prison, and after 'doing his time', returns to society after 2 years.

**Manipulation: MIPost**

Jeff is a 25 year old man on trial for pushing a stranger off a pedestrian bridge, causing permanent nerve damage and paralysis. 

Jeff enters an insanity plea. At his trial, expert doctors claim Jeff suffers from ongoing psychosis, caused by schizophrenia.  At the time of the crime, Jeff believed that the stranger was an evil villain, and angels convinced Jeff that he needed to push the man in order to save the world.

The judge rules that Jeff is not guilty by rule of insanity.  Jeff is distressed and remorseful about what he has done.

Jeff is sent to a psychiatric ward, and after 'doing his time', returns to society after 2 years.

**Responsibility**

To what extent is Jeff morally responsible for harming the victim?

| Not at all responsible 1 | 2 | 3 | 4 | Extremely responsible 5 |
| --- | --- | --- | --- | --- |
| O | O | O | O | O |

**Blame**

How much blame does Jeff deserve for his actions?

| Not blame at all 1 | 2 | 3 | 4 | Extreme blame 5 |
| --- | --- | --- | --- | --- |
| O | O | O | O | O |

**Punitiveness**

How much punishment does Jeff deserve for what he did?

| No punishment at all 1 | 2 | 3 | 4 | Extreme punishment 5 |
| --- | --- | --- | --- | --- |
| O | O | O | O | O |

**Detainment**

For how long do you think Jeff should be removed from society? (e.g., in a prison or psychiatric ward)

O Number of years: ________________________________________________

O OR: Jeff should not be formally removed from society

**General moral agency**

In general, to what extent is Jeff...

|  | Not at all 1 | 2 | 3 | 4 | Extremely 5 |
| --- | --- | --- | --- | --- | --- |
| Intentional | O | O | O | O | O |
| Willful | O | O | O | O | O |

To what extent do you think Jeff possesses the following characteristics?

|  | Not at all 1 | 2 | 3 | 4 | Extremely 5 |
| --- | --- | --- | --- | --- | --- |
| Can freely choose his own actions | O | O | O | O | O |
| Understands the effects of his behaviour on others | O | O | O | O | O |
| Knows the difference between right and wrong | O | O | O | O | O |

**Inhibitive agency**

Jeff is able to inhibit negative impulses

| Definitely not 1 | 2 | 3 | 4 | Definitely yes 5 |
| --- | --- | --- | --- | --- |
| O | O | O | O | O |

Jeff would normally refuse to participate in immoral behaviour

| Definitely not 1 | 2 | 3 | 4 | Definitely yes 5 |
| --- | --- | --- | --- | --- |
| O | O | O | O | O |

Jeff has the power to refrain from hurting others

| Definitely not 1 | 2 | 3 | 4 | Definitely yes 5 |
| --- | --- | --- | --- | --- |
| O | O | O | O | O |

**Dangerousness**

|  | Not at all 1 | 2 | 3 | 4 | Extremely 5 |
| --- | --- | --- | --- | --- | --- |
| How likely do you think it is that Jeff will commit another crime in his lifetime? | O | O | O | O | O |
| How dangerous is Jeff currently? | O | O | O | O | O |
| How comfortable would you feel attending the same public function as this man (e.g.: seeing a movie, going to an arts festival, etc.) in the future? | O | O | O | O | O |

**Rights as citizen**

In many states, people who have committed serious crimes are not allowed to vote. In your opinion, should Jeff be allowed to vote?

| Definitely not 1 | 2 | 3 | 4 | Definitely yes 5 |
| --- | --- | --- | --- | --- |
| O | O | O | O | O |

Should Jeff be allowed to own property?

| Definitely not 1 | 2 | 3 | 4 | Definitely yes 5 |
| --- | --- | --- | --- | --- |
| O | O | O | O | O |

Should Jeff be allowed to marry?

| Definitely not 1 | 2 | 3 | 4 | Definitely yes 5 |
| --- | --- | --- | --- | --- |
| O | O | O | O | O |

**Access to services**

Should the state pay for the following services for Jeff?

|  | Definitely not 1 | 2 | 3 | 4 | Definitely yes 5 |
| --- | --- | --- | --- | --- | --- |
| GED classes | O | O | O | O | O |
| Financial Advising | O | O | O | O | O |
| Career Services | O | O | O | O | O |
| Welfare Payments | O | O | O | O | O |

**Moral standing**

How important would it be to protect Jeff from harm?

| Not at all  1 | 2 | 3 | 4 | Extremely  5 |
| --- | --- | --- | --- | --- |
| O | O | O | O | O |

Jeff deserves to be treated with care and compassion

| Not at all  1 | 2 | 3 | 4 | Extremely  5 |
| --- | --- | --- | --- | --- |
| O | O | O | O | O |

Harming Jeff would be morally wrong

| Not at all  1 | 2 | 3 | 4 | Extremely  5 |
| --- | --- | --- | --- | --- |
| O | O | O | O | O |

If Jeff gets sick, he should have the right to join the registry for an organ donation

| Not at all  1 | 2 | 3 | 4 | Extremely  5 |
| --- | --- | --- | --- | --- |
| O | O | O | O | O |

**Demographics**

Please answer the following questions about yourself.

Age: _________________

Gender: _________________

Politically, I would call myself:

| Strongly Liberal | Moderately Liberal | Somewhat Liberal | Neutral | Somewhat conservative | Moderately conservative | Strongly conservative |
| --- | --- | --- | --- | --- | --- | --- |
| O | O | O | O | O | O | O |

**Attention checks**

In the vignette, Jeff

- Admitted guilt
- Was not guilty by reason of insanity

How are you feeling right now?  Many times in psychology surveys, researchers are interested in feelings.  However, in this question, we are only interested in whether you are reading the instructions.  In the following question, only select inspired.
 
How are you feeling right now?

- excited
- irritated
- happy
- frustrated
- inspired
- tired

***Other DVs***

**Rehabilitation**

Jeff is suitable for an educational rehab programme that aims to make clear the reasons his conduct was inappropriate (Not at all … Extremely)

**Perceived legitimacy**

Do you reject the legitimacy or sincerity of Jeff's insanity defense? (Not at all, it seems legitimate … Completely reject the defense)

**Patiency**

In general, to what extent is Jeff...
- Sensitive to pain (Not at all … Extremely)
- Vulnerable (Not at all … Extremely)
- Easily victimised (Not at all … Extremely)

**Proactive agency**

Jeff has the desire to do good in the world (Not at all … Extremely)
Jeff would actively try to do the right thing, even if doing so involved a personal cost (Not at all … Extremely)
Jeff feels a personal obligation to protect the welfare of others (Not at all … Extremely)

**Freedom restrictions**

After Jeff returns to society, he may still have restrictions imposed on his freedom on an ongoing basis. Indicate to what extent you believe the following sanctions should apply to Jeff:
- Jeff must report regularly to a parole officer (Definitely not … Definitely yes)
- Jeff must apply to authorities if he wishes to travel out of the state (Definitely not … Definitely yes)
- Police can track Jeff's whereabouts via GPS on his mobile phone (Definitely not … Definitely yes)
- Police can notify community members that Jeff lives in their neighbourhood (Definitely not … Definitely yes)
- Jeff's assets can be confiscated as a form of victim compensation (Definitely not … Definitely yes)
- Jeff must disclose his criminal record when applying for a job (Definitely not … Definitely yes)

# Study 2 Materials

**Manipulation: ControlPre**

Jeff is a 25 year old man, on trial for pushing a stranger off a pedestrian bridge, causing permanent nerve damage and paralysis.   

Jeff enters a guilty plea, and admits full guilt. Jeff is distressed and remorseful about what he has done.

**Manipulation: MIPre**

Jeff is a 25 year old man on trial for pushing a stranger off a pedestrian bridge, causing permanent nerve damage and paralysis. 

Jeff enters an insanity plea. At his trial, expert doctors claim Jeff suffers from ongoing psychosis, caused by schizophrenia.  At the time of the crime, Jeff believed that the stranger was an evil villain, and angels convinced Jeff that he needed to push the man in order to save the world.

The judge rules that Jeff is not guilty by rule of insanity.  Jeff is distressed and remorseful about what he has done.

**Manipulation: ControlPost**

Jeff is a 25 year old man, on trial for pushing a stranger off a pedestrian bridge, causing permanent nerve damage and paralysis.   

Jeff enters a guilty plea, and admits full guilt. Jeff is distressed and remorseful about what he has done.

Jeff is sent to a prison. After 2 years he completes his period of detainment. 

Before release, he has a hearing to review his progress. During the hearing, prison officers and psychologists from the prison testify that Jeff is eligible for release.

Having 'done his time', Jeff returns to society.

**Manipulation: MI-RecoveredPost**

Jeff is a 25 year old man on trial for pushing a stranger off a pedestrian bridge, causing permanent nerve damage and paralysis. 

Jeff enters an insanity plea. At his trial, expert doctors claim Jeff suffers from ongoing psychosis, caused by schizophrenia.  At the time of the crime, Jeff believed that the stranger was an evil villain, and angels convinced Jeff that he needed to push the man in order to save the world.

The judge rules that Jeff is not guilty by rule of insanity.  Jeff is distressed and remorseful about what he has done.

Jeff is sent to a psychiatric ward. After 2 years he completes his period of detainment.  

Before release, he has a hearing to review his progress. During the hearing, wardens and psychologists from the ward testify that Jeff is eligible for release. They state that Jeff is in control of his illness and has learned to manage his illness independently. They also testify that Jeff has developed the capacity to control his emotions and impulsive behaviors. 
 
Having 'done his time', Jeff returns to society.

**Manipulation: MI-UnrecoveredPost**

Jeff is a 25 year old man on trial for pushing a stranger off a pedestrian bridge, causing permanent nerve damage and paralysis. 

Jeff enters an insanity plea. At his trial, expert doctors claim Jeff suffers from ongoing psychosis, caused by schizophrenia.  At the time of the crime, Jeff believed that the stranger was an evil villain, and angels convinced Jeff that he needed to push the man in order to save the world.

The judge rules that Jeff is not guilty by rule of insanity.  Jeff is distressed and remorseful about what he has done.

Jeff is sent to a psychiatric ward. After 2 years he completes his period of detainment. 

Before release, he has a hearing to review his progress. During the hearing, wardens and psychologists from the ward testify that Jeff is eligible for release. They state that he could, however, learn to be more in control of his illness and to better manage his illness independently. They also testify that Jeff has struggled at times to control his emotions and impulsive behaviours.
 
Having 'done his time', Jeff returns to society.

**Responsibility**

To what extent is Jeff morally responsible for harming the victim?

| Not at all responsible 1 | 2 | 3 | 4 | Extremely responsible 5 |
| --- | --- | --- | --- | --- |
| O | O | O | O | O |

**Blame**

How much blame does Jeff deserve for his actions?

| Not blame at all 1 | 2 | 3 | 4 | Extreme blame 5 |
| --- | --- | --- | --- | --- |
| O | O | O | O | O |

**Punitiveness**

How much punishment does Jeff deserve for what he did?

| No punishment at all 1 | 2 | 3 | 4 | Extreme punishment 5 |
| --- | --- | --- | --- | --- |
| O | O | O | O | O |

**Detainment**

For how long do you think Jeff should be removed from society? (e.g., in a prison or psychiatric ward)

O Number of years: ________________________________________________

O OR: Jeff should not be formally removed from society

**General moral agency**

To what extent do you think Jeff possesses the following characteristics?

|  | Not at all 1 | 2 | 3 | 4 | Extremely 5 |
| --- | --- | --- | --- | --- | --- |
| Mental capacity to think about and plan for the future | O | O | O | O | O |
| Exercise self-control | O | O | O | O | O |
| Can freely choose his own actions | O | O | O | O | O |
| Knows the difference between right and wrong | O | O | O | O | O |
| Understands the effects of his behaviour on others | O | O | O | O | O |
| Is aware of social norms | O | O | O | O | O |

**Inhibitive agency**

Jeff is able to inhibit negative impulses

| Definitely not 1 | 2 | 3 | 4 | Definitely yes 5 |
| --- | --- | --- | --- | --- |
| O | O | O | O | O |

Jeff has the capacity to resist doing the wrong thing

| Definitely not 1 | 2 | 3 | 4 | Definitely yes 5 |
| --- | --- | --- | --- | --- |
| O | O | O | O | O |

Jeff has the power to refrain from hurting others

| Definitely not 1 | 2 | 3 | 4 | Definitely yes 5 |
| --- | --- | --- | --- | --- |
| O | O | O | O | O |

**Dangerousness**

|  | Not at all 1 | 2 | 3 | 4 | Extremely 5 |
| --- | --- | --- | --- | --- | --- |
| How likely do you think it is that Jeff will commit another crime in his lifetime? | O | O | O | O | O |
| How dangerous is Jeff currently? | O | O | O | O | O |
| How comfortable would you feel attending the same public function as this man (e.g.: seeing a movie, going to an arts festival, etc.) in the future? | O | O | O | O | O |

**Rights as citizen**

In many states, people who have committed serious crimes are not allowed to vote. In your opinion, should Jeff be allowed to vote?

| Definitely not 1 | 2 | 3 | 4 | Definitely yes 5 |
| --- | --- | --- | --- | --- |
| O | O | O | O | O |

Should Jeff be allowed to own property?

| Definitely not 1 | 2 | 3 | 4 | Definitely yes 5 |
| --- | --- | --- | --- | --- |
| O | O | O | O | O |

Should Jeff be allowed to marry?

| Definitely not 1 | 2 | 3 | 4 | Definitely yes 5 |
| --- | --- | --- | --- | --- |
| O | O | O | O | O |

**Access to services**

Should the state pay for the following services for Jeff?

|  | Definitely not 1 | 2 | 3 | 4 | Definitely yes 5 |
| --- | --- | --- | --- | --- | --- |
| GED classes | O | O | O | O | O |
| Financial Advising | O | O | O | O | O |
| Career Services | O | O | O | O | O |
| Welfare Payments | O | O | O | O | O |

**Moral standing**

How important would it be to protect Jeff from harm?

| Not at all  1 | 2 | 3 | 4 | Extremely  5 |
| --- | --- | --- | --- | --- |
| O | O | O | O | O |

Jeff deserves to be treated with care and compassion

| Not at all  1 | 2 | 3 | 4 | Extremely  5 |
| --- | --- | --- | --- | --- |
| O | O | O | O | O |

Harming Jeff would be morally wrong

| Not at all  1 | 2 | 3 | 4 | Extremely  5 |
| --- | --- | --- | --- | --- |
| O | O | O | O | O |

If Jeff gets sick, he should have the right to join the registry for an organ donation

| Not at all  1 | 2 | 3 | 4 | Extremely  5 |
| --- | --- | --- | --- | --- |
| O | O | O | O | O |

**Demographics**

Please answer the following questions about yourself.

Age: _________________

Gender: _________________

Politically, I would call myself:

| Strongly Liberal | Moderately Liberal | Somewhat Liberal | Neutral | Somewhat conservative | Moderately conservative | Strongly conservative |
| --- | --- | --- | --- | --- | --- | --- |
| O | O | O | O | O | O | O |

**Attention checks**

In the vignette, Jeff

- Admitted guilt
- Was not guilty by reason of insanity

In the vignette, wardens and psychologists testified at Jeff’s hearing that Jeff:

- Had learned to control his illness and impulses
- Had struggled to control his illness and impulses

How are you feeling right now?  Many times in psychology surveys, researchers are interested in feelings.  However, in this question, we are only interested in whether you are reading the instructions.  In the following question, only select inspired.
 
How are you feeling right now?

- excited
- irritated
- happy
- frustrated
- inspired
- tired

***Other DVs***

**Perceived legitimacy**

Do you reject the legitimacy or sincerity of Jeff's insanity defense? (Not at all, it seems legitimate … Completely reject the defense)

**Freedom restrictions**

After Jeff returns to society, he may still have restrictions imposed on his freedom on an ongoing basis. Indicate to what extent you believe the following sanctions should apply to Jeff:
- Jeff must report regularly to a parole officer (Definitely not … Definitely yes)
- Jeff must apply to authorities if he wishes to travel out of the state (Definitely not … Definitely yes)
- Police can track Jeff's whereabouts via GPS on his mobile phone (Definitely not … Definitely yes)
- Police can notify community members that Jeff lives in their neighbourhood (Definitely not … Definitely yes)
- Jeff's assets can be confiscated as a form of victim compensation (Definitely not … Definitely yes)
- Jeff must disclose his criminal record when applying for a job (Definitely not … Definitely yes)

# Study 3 Materials (Registered Report)

**Attention check**

How are you feeling right now?  Many times in psychology surveys, researchers are interested in feelings.  However, in this question, we are only interested in whether you are reading the instructions.  In the following question, only select inspired.
 
How are you feeling right now?

- excited
- irritated
- happy
- frustrated
- inspired
- tired

**Manipulation: Control, Robbery**

Last year, Liam faced charges for robbery. He entered a gas station and threatened the cashier, telling him he had a weapon. He then stole several hundred dollars.    

Liam entered a guilty plea, and admitted full guilt in court. He testified that he was motivated by financial reasons. He was distressed and remorseful about what he had done.

The judge ruled that he should serve 12 months in prison.

Twelve months pass, and Liam completes his order. Having done his time, he returns to society.

**Manipulation: MI (bipolar), Robbery**

Last year, Liam faced charges for robbery. He entered a gas station and threatened the cashier, telling him he had a weapon. He then stole several hundred dollars.    

Liam entered an insanity plea in court. At his trial, doctors testified that Liam has bipolar disorder. At the time of the crime, he was going through a severe manic phase involving hyperactivity and delusions. He was distressed and remorseful about what he had done.

The judge ruled that Liam was not guilty by reason of insanity, and should be sent to a psychiatric ward for 12 months.

Twelve months pass, and Liam completes his order. Having done his time, he returns to society.

**Manipulation: MI (brain injury), Robbery**

Last year, Liam faced charges for robbery. He entered a gas station and threatened the cashier, telling him he had a weapon. He then stole several hundred dollars.    

Liam entered an insanity plea in court. At his trial, doctors testified that Liam has an acquired brain injury. At the time of the crime, he was suffering from hyperactivity and delusions. He was distressed and remorseful about what he had done.

The judge ruled that Liam was not guilty by reason of insanity, and should be sent to a psychiatric ward for 12 months.

Twelve months pass, and Liam completes his order. Having done his time, he returns to society.

**Manipulation: Control, sexual offence**

Last year, Liam faced charges for a sexual offence. He exposed himself at a public park in front of several women.    

Liam entered a guilty plea, and admitted full guilt in court. He testified that he did it to get sexual satisfaction. He was distressed and remorseful about what he had done.

The judge ruled that he should spend 12 months on a community-based supervision order.

Twelve months pass, and Liam completes his order. Having done his time, he returns to society.

**Manipulation: MI (bipolar), sexual offence**

Last year, Liam faced charges for a sexual offence. He exposed himself at a public park in front of several women.    

Liam entered an insanity plea in court. At his trial, doctors testified that Liam has bipolar disorder. At the time of the crime, he was going through a severe manic phase involving hyperactivity and delusions. He was distressed and remorseful about what he had done.

The judge ruled that Liam was not guilty by reason of insanity, and should spend 12 months undergoing community-based psychological treatment.

Twelve months pass, and Liam completes his order. Having done his time, he returns to society.

**Manipulation: MI (brain injury), sexual offence**

Last year, Liam faced charges for a sexual offence. He exposed himself at a public park in front of several women.    

Liam entered an insanity plea in court. At his trial, doctors testified that Liam has bipolar disorder. Liam entered an insanity plea in court. At his trial, doctors testified that Liam has acquired brain injury. At the time of the crime, he was suffering from hyperactivity and delusions. He was distressed and remorseful about what he had done.

The judge ruled that Liam was not guilty by reason of insanity, and should spend 12 months undergoing community-based psychological treatment.

Twelve months pass, and Liam completes his order. Having done his time, he returns to society.

**Inhibitive agency**

Liam is able to inhibit negative impulses

| Definitely not 1 | 2 | 3 | 4 | Definitely yes 5 |
| --- | --- | --- | --- | --- |
| O | O | O | O | O |

Liam has the capacity to resist doing the wrong thing

| Definitely not 1 | 2 | 3 | 4 | Definitely yes 5 |
| --- | --- | --- | --- | --- |
| O | O | O | O | O |

Liam has the power to refrain from hurting others

| Definitely not 1 | 2 | 3 | 4 | Definitely yes 5 |
| --- | --- | --- | --- | --- |
| O | O | O | O | O |

**Patiency**

In general, to what extent is Liam...

|  | Not at all 1 | 2 | 3 | 4 | Extremely 5 |
| --- | --- | --- | --- | --- | --- |
| …vulnerable to mistreatment | O | O | O | O | O |
| …vulnerable to being harmed | O | O | O | O | O |
| …vulnerable to victimization | O | O | O | O | O |

**Dangerousness**

|  | Not at all 1 | 2 | 3 | 4 | Extremely 5 |
| --- | --- | --- | --- | --- | --- |
| How likely do you think it is that Liam will commit another crime in his lifetime? | O | O | O | O | O |
| How dangerous is Liam currently? | O | O | O | O | O |
| How comfortable would you feel attending the same public function as this man (e.g.: seeing a movie, going to an arts festival, etc.) in the future? | O | O | O | O | O |

**Rights as citizen**

In many states, people who have committed serious crimes are not allowed to vote. In your opinion, should Liam be allowed to vote?

| Definitely not 1 | 2 | 3 | 4 | Definitely yes 5 |
| --- | --- | --- | --- | --- |
| O | O | O | O | O |

Should Liam be allowed to own property?

| Definitely not 1 | 2 | 3 | 4 | Definitely yes 5 |
| --- | --- | --- | --- | --- |
| O | O | O | O | O |

Should Liam be allowed to marry?

| Definitely not 1 | 2 | 3 | 4 | Definitely yes 5 |
| --- | --- | --- | --- | --- |
| O | O | O | O | O |

**Access to services**

Should the state pay for the following services for Liam?

|  | Definitely not 1 | 2 | 3 | 4 | Definitely yes 5 |
| --- | --- | --- | --- | --- | --- |
| GED classes | O | O | O | O | O |
| Financial Advising | O | O | O | O | O |
| Career Services | O | O | O | O | O |
| Welfare Payments | O | O | O | O | O |

**Moral standing**

How important would it be to protect Liam from harm?

| Not at all  1 | 2 | 3 | 4 | Extremely  5 |
| --- | --- | --- | --- | --- |
| O | O | O | O | O |

Liam deserves to be treated with care and compassion

| Not at all  1 | 2 | 3 | 4 | Extremely  5 |
| --- | --- | --- | --- | --- |
| O | O | O | O | O |

Harming Liam would be morally wrong

| Not at all  1 | 2 | 3 | 4 | Extremely  5 |
| --- | --- | --- | --- | --- |
| O | O | O | O | O |

If Liam gets sick, he should have the right to join the registry for an organ donation

| Not at all  1 | 2 | 3 | 4 | Extremely  5 |
| --- | --- | --- | --- | --- |
| O | O | O | O | O |

**Demographics**

Please answer the following questions about yourself.

Age: _________________

Gender: _________________

Politically, I would call myself:

| Strongly Liberal | Moderately Liberal | Somewhat Liberal | Neutral | Somewhat conservative | Moderately conservative | Strongly conservative |
| --- | --- | --- | --- | --- | --- | --- |
| O | O | O | O | O | O | O |
|  |  |  |  |  |  |  |

**Attention check**

In the vignette, Liam

- Admitted guilt
- Was not guilty by reason of insanity

# Additional Measures in Studies 1 and 2

Studies 1 and 2 also included a single-item measure of the perceived legitimacy of the defense (displayed to those in the MI at trial condition) at the end of the survey, in order to provide a possible explanation in the case that there were no main effects of the defense on responsibility. This was not necessary.

Study 1 also included a single-item measure of suitability for rehabilitation, which was included to explore whether participants thought mentally ill defendants deserved *non-punitive* criminal justice responses (at trial). In fact, there was no difference between groups on this measure, *t*(162)=0.11, *p*=.912.

We also included a measure of proactive agency (the ability to actively engage in moral behaviors) in Study 1, displayed to all participants. This measure showed poor convergent validity with the general agency scale (*r*=-.12, *p*=.026) and was removed from the survey battery.

Moreover, we included a three-item patiency scale in Study 1. We had considered whether any attributions of reduced agency might be indirect, simply due to increased patiency signaled by the mental illness defense, since the two are normally inversely correlated. There was some relationship between general agency and patiency (*r*=-.39, *p*<.001), but not between inhibitive agency and patiency (*r*=.09, *p*=.089); and main effects of the defense were greater on agency than they were on patiency. We had therefore dropped the measure from Study 2.

We also included a fourth rights measure in both Studies 1 and 2, pertaining to freedom restrictions (e.g., freedom of movement). Results for this outcome were in line with our model, in that the defense was associated with greater freedom restrictions via inhibitive agency and dangerousness (e.g., in Study 1, *B_indirect_*=.12, *SE=*.04, 99% CI [.04, .27]). Following peer feedback, we deemed this measure to be a relatively weak test of our hypothesis, since people with mental illness are in fact sometimes subject (by law) to freedom restrictions even following release from psychiatric detainment. The three remaining rights measures are broad, universal rights that allow for a stricter test of our hypothesis.

# Study 1 Exploratory Analysis: SEM Results

We conducted path analysis using maximum likelihood estimation in AMOS (v.26), to assess if MI condition predicted rights, access to services, and moral standing via two separate indirect paths. First, via inhibitive agency and dangerousness (serial mediation). Second, via patiency. We conducted three models testing each of the dependent variables. We used boostrapping procedures with 1000 resamples to calculate 98.33% bias corrected confidence intervals (adjusted for three dependent rights variables) for all regression coefficients. Model statistics are provided in the figures that follow.

The results followed the same pattern for all three models. There were significant indirect effects of MI on each dependent variable via inhibitive agency and dangerousness. Specifically, participants in the MI condition perceived the protagonist as having less inhibitive agency, which in turn predicted greater perceptions of dangerousness, and fewer rights, access to services and lower moral standing.

The same pattern of results was also found for all three models in relation to patiency. There was a significant, positive indirect effect of MI condition via patiency on each of the dependent variables. That is, people in the MI condition perceived Jeff as higher in patiency, and in turn more deserving of rights, access to services and higher in moral standing.

The model fit indices, while poor, are not relevant to our analysis. We are not interested in explaining variance and relationships between all variables in the structural models, but rather, the exact paths specified by our theoretic model.

MI Condition

(0=NonMID, 1=MID)

Inhibitive Agency

Dangerousness

Moral Standing

Patiency

-0.46

[-0.75, -0.16]

*p* = .001

-1.24

[-3.12, -0.53]

*p* = .001

0.28

[-0.23, 0.75]

*p* = .196

(direct effect)

-0.36

[-0.58, -0.12]

*p* = .001

0.75

[0.44, 1.07]

*p* = .001

0.22

[-0.02, 0.48]

*p* = .028

.56

*1.12, p = .002*

*0.69, p <.001*

*0.67, p < .001*

*0.73, p < .001*

There is a significant, negative indirect effect of MI condition on moral standing, via inhibitive agency and dangerousness, *B*=-0.21, [-0.44, -0.06], *SE*=0.08, *p*=.001.

There is a significant, positive indirect effect of MI condition on moral standing, via patiency, *B*=0.17, [0.01, 0.41], *SE*=0.10, *p*=.024.

Model fit indices: χ^2^=17.54, *p*=.001; RMSEA=.17, pclose=.004; TLI=.64.

MI Condition

(0=NonMID, 1=MID)

Inhibitive Agency

Dangerousness

Services

Patiency

-0.46

[-0.75, -0.16]

*p* = .001

-1.24

[-3.12, -0.53]

*p* = .001

.33

[-0.17, 0.82]

*p* = .111

(direct effect)

-0.35

[-0.59, -0.09]

*p* = .002

0.75

[0.44, 1.07]

*p* = .001

0.37

[0.08, 0.64]

*p* = .002

.56

*1.12, p = .002*

*0.69, p <.001*

*0.67, p < .001*

*1.54, p < .001*

There is a significant, negative indirect effect of MI condition on services, via inhibitive agency and dangerousness, *B*=-0.20, [-0.43, -0.05], *SE*=0.08, *p*=.001.

There is a significant, positive indirect effect of MI condition on services, via patiency, *B*=0.28, [0.08, 0.56], *SE*=0.10, *p*=.001.

Model fit indices: χ^2^=17.51, *p*=.001; RMSEA=.17, pclose=.004; TLI=.64.

MI Condition

(0=NonMID, 1=MID)

Inhibitive Agency

Dangerousness

Rights as Citizen

Patiency

-0.46

[-0.75, -0.16]

*p* = .001

-1.24

[-3.12, -0.53]

*p* = .001

0.33

[-0.69, 0.04]

*p* = .034

(direct effect)

-0.60

[-0.79, -0.42]

*p* = .001

0.75

[0.44, 1.07]

*p* = .001

0.23

[0.004, 0.45]

*p* = .015

.56

*1.12, p = .002*

*0.69, p <.001*

*0.67, p < .001*

*0.74, p < .001*

There is a significant, negative indirect effect of MI condition on rights as citizen, via inhibitive agency and dangerousness, *B*=-0.35, [-0.59, -0.12], *SE*=0.10, *p*=.001.

There is a significant, positive indirect effect of MI condition on rights as citizen, via patiency, *B*=0.17, [0.01, 0.37], *SE*=0.07, *p*=.013.

Model fit indices: χ^2^=21.10, *p*<.001; RMSEA=.19, pclose=.001; TLI=.67.
